# Multiple ‘Brown Tumors’ Masquerading as Metastatic Bone Disease

**DOI:** 10.7759/cureus.431

**Published:** 2015-12-23

**Authors:** Raju Vaishya, Amit Kumar Agarwal, Harsh Singh, Vipul Vijay

**Affiliations:** 1 Orthopaedics, Indraprastha Apollo Hospitals

**Keywords:** brown tumor, osteitis fibrosa cystica, von recklinghausen’s disease

## Abstract

‘Brown tumors’ are known as ‘osteitis fibrosa cystica’ or ‘Von Recklinghausen’s disease’ of the bone. A high index of suspicion is required by the treating doctor for diagnosing a ‘brown tumor’ in its early stage. Clinical suspicion, along with laboratory and radiological investigations, is required to diagnose this condition. We present a case of a 65-year-old woman who had multiple bony lesions and a thyroid nodule, which was initially considered as a metastatic bone disease, but later turned out to be ‘brown tumors.' In all cases with multiple osteolytic lesions, a possibility of ‘brown tumor’ must be kept in mind.

## Introduction

‘Brown tumor’ is a rare manifestation of prolonged hyperparathyroidism (primary, secondary, or tertiary) and has always been a subject of tremendous interest in orthopaedics [1-2]. The incidence of these tumors has more commonly been seen in patients with chronic kidney disease on dialysis who have repressed ability to convert 25 hydroxycholecalciferol to 1,25 dihydroxy-cholecalciferol, resulting in prolonged hyperparathyroidism [3-4]. Its name derives from the fact that there is s characteristic brown colouration due to haemosiderin deposition into the bone cysts because of osteolysis. Diagnosis of 'brown tumor' is often challenging clinically and, hence, a high index of suspicion is essential to make a diagnosis. Radiographically, these lesions initially present as lytic lesions of the bone (classical 'salt and pepper' appearance and 'ground glass' appearance have also been described), which, upon treatment of the underlying cause, regress to appear as areas of increased radiodensity. Surgical biopsy is a gold standard in the diagnosis, but radiological findings and biochemical tests, including serum parathyroid hormone (PTH), vitamin D level, etc., help in making the diagnosis. Treatment of these tumors consists mainly of partial or complete resection of the parathyroid gland. In most cases, curettage is performed before an attempt to reach a precise diagnosis is made, and this leads to unnecessary bony resection. The tumor undergoes spontaneous regression after addressing the parathyroid cause. The pattern of regression is a subject of further study and has less clearly been defined in existing literature [5]. We present a case of a 65-year-old woman who had multiple bony lesions and a thyroid nodule, which were initially considered to be metastatic bone disease, but later turned out to be ‘brown tumor'.

## Case presentation

A 65-year-old female presented with complaints of inability to bear weight on both the lower limbs, low back pain, and pain in both thighs for the last six months. There was no history of fever, weight loss, trauma, or any other constitutional symptoms. She reported having a painless, non-increasing lump in the neck for last 23 years. When she became bedridden due to pain in back and thighs, she presented to our hospital for further management. The patient underwent a thorough clinical examination and was found to have tenderness in lumbar vertebrae, chest, and mid-thighs with an intact neurological status. 

**Figure 1:**
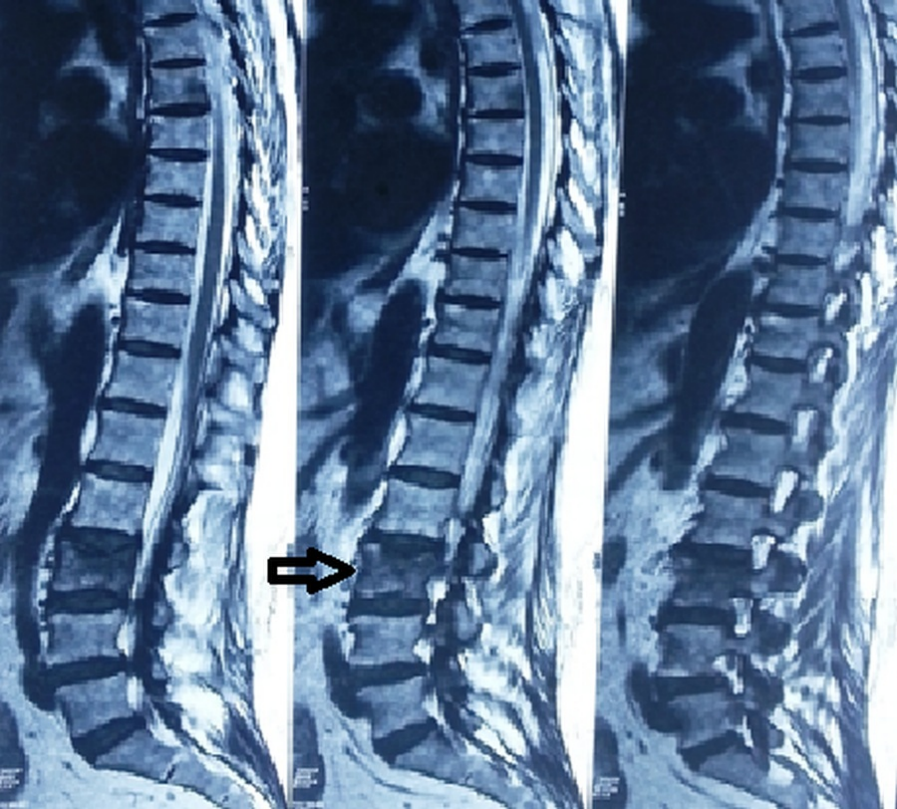
MRI fat suppression image of lumbar spine showing compression fracture of the L-3 vertebra with altered bone marrow signals (Arrow-marked).

Before presenting to us, the patient had undergone multiple diagnostic investigations. A magnetic resonance imaging (MRI) of the lumbar spine showed a compression fracture of the L-3 vertebral body with altered bone marrow signals (Figure 1).

**Figure 2:**
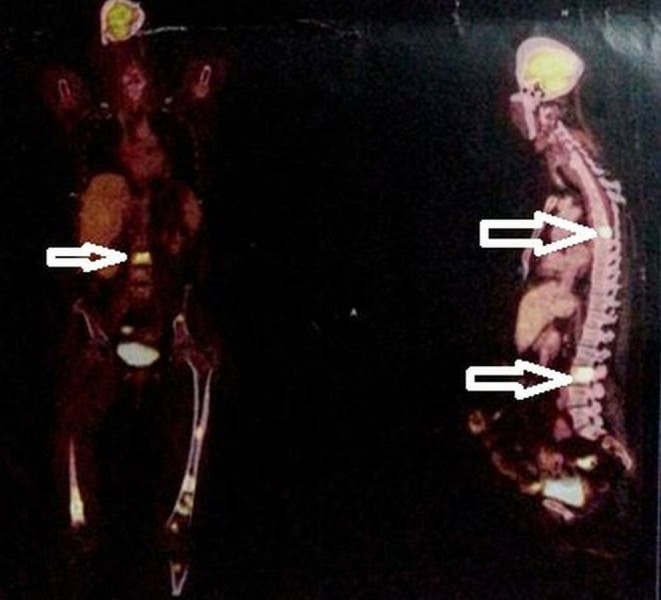
PET scan images showing a hypermetabolic lesion in the right lobe of the thyroid suggestive of a primary malignancy in the thyroid with metastasis to the liver (Arrow-marked).

The positron emission tomogram (PET) scan revealed a hypermetabolic lesion in the right lobe of the thyroid (suggestive of a thyroid malignancy) with multiple bony metastases to the pelvis, third lumbar, seventh dorsal vertebral body, ribs, manubrium sternum, and liver (Figure 2).

**Figure 3:**
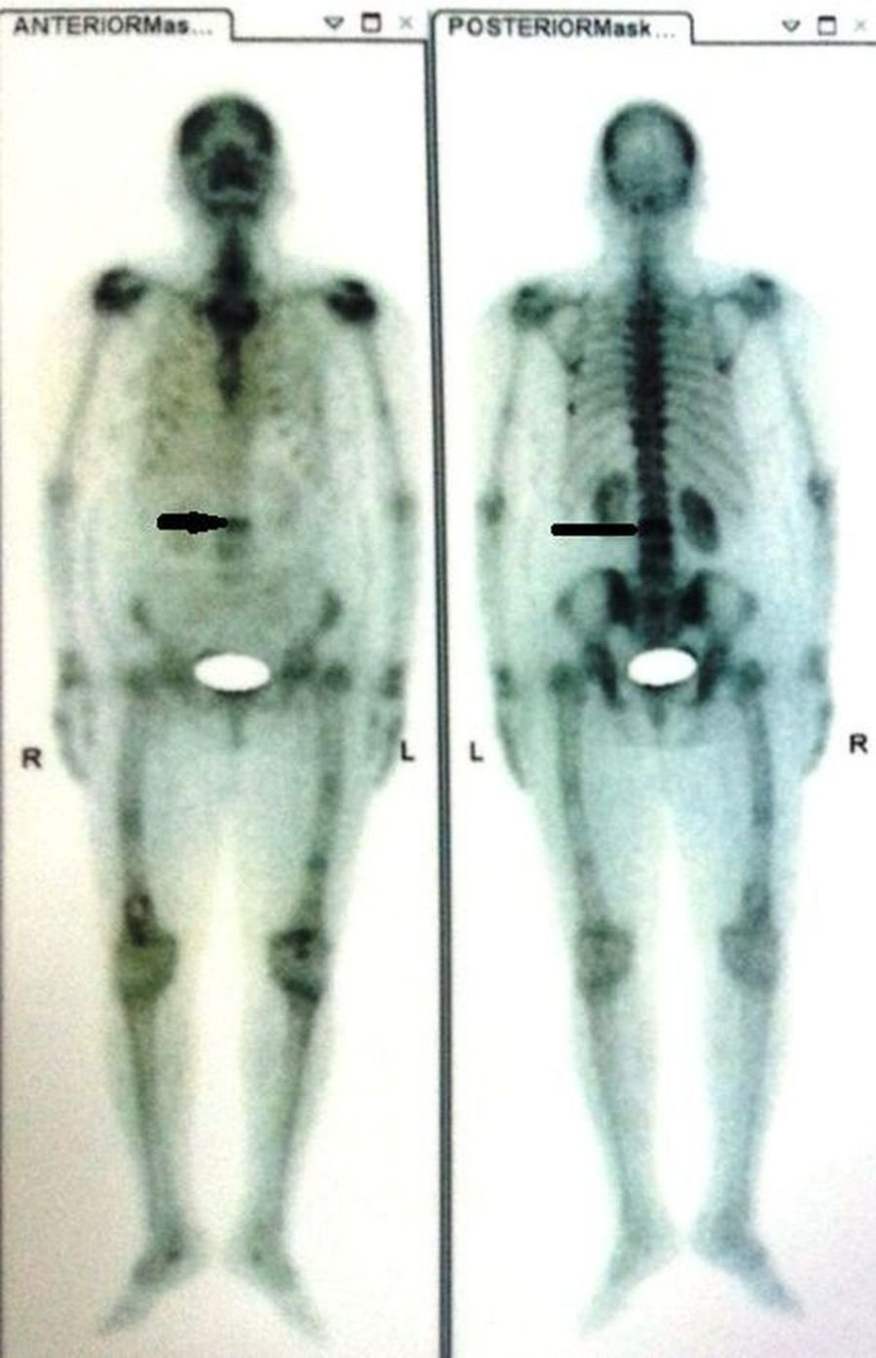
Tc99 bone scan image showing increased uptake at multiple sites, including the bilateral humerus, left acetabulum, spine, femur, and ribs

The technetium isotope (Tc) 99 bone scan confirmed this increased uptake in all phases at multiple sites (Figure 3).

She had a history of biopsies of the sternoclavicular joint and right femoral shaft, which were the hot spots of the isotope bone scan. Both of these biopsies were inconclusive and showed no signs of a pathological disease. A fine needle aspiration cytology of the right lobe of thyroid ruled out the possibility of malignancy as was suspected on PET scan.

**Figure 4:**
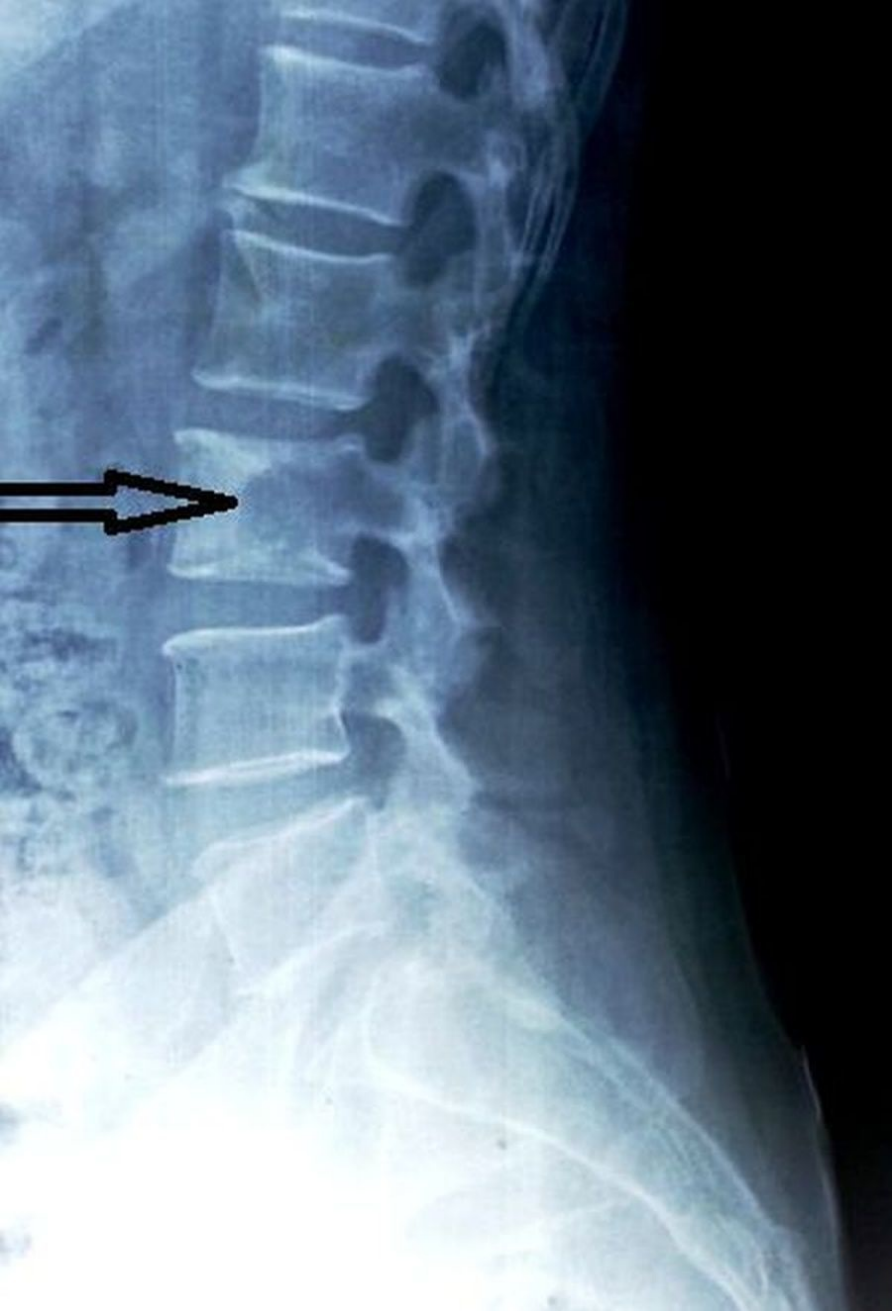
Plain radiograph, lateral view of the lumbosacral spine showing an osteolytic lesion in the L-3 vertebral body.

At admission, we investigated this patient further for serum Vitamin D and PTH levels. The serum 25-hydroxy Vitamin D level was abnormally low < 3 ng/ml (normal values: 20-32 ng/ml), and there was almost a 40-fold rise in the PTH levels (924.7 pg/ml). A plain radiograph of the lumbosacral spine (Figure 4) revealed an osteolytic lesion of the L3 vertebral body. Based on these test results and clinical features, a diagnosis of ‘brown tumor’ due to secondary hyperparathyroidism (arising from severe Vitamin D deficiency) was made. Informed patient consent was obtained. No identifying patient information is disclosed in this study.

After diagnosing, we started her on injection calcitriol - 6,00,000 units intramuscular as a single dose, calcium carbonate - 1.2 gm twice a day, and cholecalciferol - 60,000 units every week for eight weeks, along with other symptomatic therapy. 

The patient responded dramatically well to high-dose Vitamin D supplementation and calcium therapy, and her biochemical parameter, including serum PTH, and serum Vitamin D came to a normal range after two months of treatment. She started walking again with the help of a walker after three weeks and the back pain was alleviated significantly.

## Discussion

Although named as a ‘tumor’, 'brown tumor' is not a true malignancy; however, it may mimic malignancy. The differential diagnosis of 'brown tumor' includes fibrous dysplasia, fibrous cortical defect, osteoblastoma, giant cell tumour, metastasis, multiple myeloma, aneurysmal bone cyst, chondroblastoma, chondromyxoid fibroma, infection, non-ossifying fibroma, enchondroma, eosinophilic granuloma, and simple bone cyst. The actual global estimate of the incidence of ‘brown tumor’ is not known. Clinically, they consist of three subtypes:

A) Primary, due to a parathyroid gland adenoma or other primary parathyroid pathologies leading to excessive PTH secretion.

B) Secondary, due to renal anomalies severely affecting its ability to manufacture and transport 1, 25 dihydroxycholecalciferol (Vitamin D-3) and variations in serum calcium and phosphorus levels.

C) Tertiary, due to a receptor level anomaly.

Awareness of this condition is necessary for early diagnosis. In patients presenting with multiple lytic lesions of the bone, 'brown tumor' (secondary to hyperparathyroidism) must be excluded. These tumors usually manifest as uni- or multilocular radiolucencies with expansion and thinning of cortices on radiographs [6]. Serum PTH level is often increased significantly, and it may be associated with low serum Vitamin D level. Microscopically, these lesions appear as multinucleated giant cells in a spindle cell matrix containing hemosiderin deposits. In precarious situations, further investigations like serum electrophoresis, CT, MRI, PET, and isotope bone scans are required to confirm the diagnosis.

Even though a biopsy is considered the gold standard for diagnosing such lesions, it is likely that a biopsy can be inconclusive in many such cases. A precise diagnosis cannot be relied upon by a single diagnostic modality, and a comprehensive picture has to be taken into account as evident in the case above. Serum PTH levels coupled with a bone scan provide a highly accurate measure to clinch a precise diagnosis in cases of ‘brown tumor’ [7].

Classically, the clinical symptoms of 'brown tumor' have been described as “bone, stone, abdominal groan, and psychic moan” [8]. These symptoms have been attributed to hypercalcemia, excessive resorption of bone, and mental agony that comes along. It affects women mostly in the fourth and fifth decades of life and, rarely, younger individuals have been seen to be affected.

There is rapid osteoclastic bone resorption resulting from the effect of PTH. Haemorrhage, vascular fibrous tissue, and granulation tissue replace the existing bone. Localized accumulation of fibrous tissues and giant cells results in bone expansion [9]. 'Brown tumors' are primarily observed in the face, mandible, and neck region, followed by the pelvis, ribs, and femur. Chances of involvement of the appendicular skeleton are more commonly seen with these tumors, and there is a rare occurrence of pathological fractures. In cases of primary hyperparathyroidism, removal the parathyroid mass offers a lucrative treatment option; the pathological fractures may need stabilization or surgical correction [10].

## Conclusions

A high index of suspicion is required to diagnose a 'brown tumor'. Multiple osteolytic lesions should raise a suspicion of a 'brown tumor'. Even though biopsy is considered to be a ‘gold standard’ to diagnose bone lesions, an inconclusive biopsy report has to be correlated with clinical parameters and other investigations.
